# Pigmentary Markers in Danes – Associations with Quantitative Skin Colour, Nevi Count, Familial Atypical Multiple-Mole, and Melanoma Syndrome

**DOI:** 10.1371/journal.pone.0150381

**Published:** 2016-03-03

**Authors:** Peter Johansen, Jeppe Dyrberg Andersen, Linnea Nørgård Madsen, Henrik Ullum, Martin Glud, Claus Børsting, Robert Gniadecki, Niels Morling

**Affiliations:** 1 Section of Forensic Genetics, Department of Forensic Medicine, Faculty of Health and Medical Sciences, University of Copenhagen, DK-2100 Copenhagen, Denmark; 2 Department of Dermatology, Faculty of Health and Medical Sciences, University of Copenhagen, Bispebjerg Hospital, Copenhagen, Denmark; 3 Department of Clinical Immunology, University Hospital Copenhagen, Rigshospitalet, Copenhagen, Denmark; Ohio State University Medical Center, UNITED STATES

## Abstract

To investigate whether pigmentation genes involved in the melanogenic pathway (melanogenesis) contributed to melanoma predisposition, we compared pigmentary genetics with quantitative skin pigmentation measurements, the number of atypical nevi, the total nevus count, and the familial atypical multiple mole and melanoma (FAMMM) syndrome. We typed 32 pigmentary SNP markers and sequenced *MC1R* in 246 healthy individuals and 116 individuals attending periodic control for malignant melanoma development, 50 of which were diagnosed with FAMMM. It was observed that individuals with any two grouped *MC1R* variants (missense, NM_002386:c. 456C > A (p.TYR152*), or NM_002386:c.83_84insA (p.Asn29Glnfs*14) had significantly (p<0.001) lighter skin pigmentation of the upper-inner arm than those with none or one *MC1R* variant. We did not observe any significant association of the *MC1R* variants with constitutive pigmentation measured on the buttock area. We hypothesize that the effect of *MC1R* variants on arm pigmentation is primarily reflecting the inability to tan when subjected to UVR. A gender specific effect on skin pigmentation was also observed, and it was found that the skin pigmentation of females on average were darker than that of males (p<0.01). We conclude that *MC1R* variants are associated with quantitative skin colour in a lightly pigmented Danish population. We did not observe any association between any pigmentary marker and the FAMMM syndrome. We suggest that the genetics of FAMMM is not related to the genetics of the pigmentary pathway.

## Introduction

Human skin colour is greatly influenced by environmental factors, including ultraviolet radiation (UVR). UVR is high in equatorial regions of the world, where darker skinned populations are found, and lower in regions distant to equator, where lighter skinned populations are found [1]. Excessive UVR of the skin induces DNA damage and is considered one of the main risk factors of developing various skin cancers. Dark skin, with high levels of the pigment eumelanin, protects against UVR, because eumelanin block the effects of UVR. On the other hand, vitamin D synthesis requires UVR and human skin colour diversity reflects the balance of letting enough UVR through the skin to produce vitamin D and preventing DNA-damage caused by UVR [2].

Melanin is produced in melanosomes, which are lysosome-related organelles present in melanocytes. Two types of melanin are produced, pheomelanin (yellow/red) and eumelanin (brown/black). Pheomelanin levels are rather constant among people with different skin colours whereas eumelanin levels vary [3]. Eumelanin is more efficient than pheomelanin in blocking UVR and in scavenging the reactive oxygen species produced from UVR of the skin (reviewed in [4]). Pheomelanin confers a carcinogenic risk in mice independently of UVR [5]. Melanocytes are found in the basal layer of the skin together with keratinocytes and the two cell types form the epidermal melanocyte unit [6]. Keratinocyte cells function as the primary barrier against environmental damage, including UVR. DNA damage in the keratinocyte induces expression of tumour protein p53 (P53), which induces melanin synthesis by increasing the level of α-melanocyte stimulating hormone (αMSH) that binds to the melanocyte-membrane-bound melanocortin 1 receptor (MC1R) and increases the cAMP level in the melanocytes. This results in increased transcription of several pigmentation genes via microphtalmia associated transcription factor (*MITF*) (reviewed in [7]).

Many of the key genes involved in the melanogenic pathway are associated with skin colour and skin cancer risk including agouti signalling protein (*ASIP*), oculocutaneous albinism 2 (*OCA2*), solute carrier family 45 member 2 (*SLC45A2*), tyrosinase (*TYR)*, tyrosinase-related protein 1 (*TYRP1)*, and melanocortin 1 receptor *MC1R* [8–17]. Variants in the *MC1R* have been studied extensively in relation to the risk of developing various skin cancers [18–21], and certain *MC1R* variants are associated with increased survival of melanoma patients [22]. Mutations in *MC1R* cause the red hair colour phenotype that is characterized by red hair, fair skin, and inability to tan [23–27]. Mutations in *MC1R* may result in either diminished α-MSH binding or decreased cAMP signalling resulting in decreased production of eumelanin.

UVR exposure is a major health risk factor, especially in light skinned populations [28,29]. Lightly pigmented skin and a large number of atypical nevi is associated with increased risk of developing malignant melanoma [11,30–33]. Atypical nevi are more prevalent in melanoma patients from light than from dark skinned populations [34]. The grade of cytological atypia of the nevi is an important risk factor for the development of malignant melanoma [35,36]. The familial atypical multiple-mole and melanoma (FAMMM) syndrome is defined by (1) occurrence of melanoma in one or more first or second degree relatives, (2) large numbers of moles (often greater than 50) some of which are atypical and often variable in size, and (3) moles that demonstrate certain distinct histologic features [37,38]. Genetic mutations have been identified in melanoma prone families including mutations in cyclin kinase 2A (*CDKN2A*) and cyclin kinase 4 (*CDK4*) genes, both of which encode proteins involved in the retinoblastoma pathway [39,40]. Genetic variants of *CDKN2A* are associated with the number and distribution of atypical nevi [41].

*MC1R* variants can modify the penetrance of *CDKN2A* mutations, as carriers of mutations in both genes are at increased risk of developing malignant melanoma compared to individuals carrying a mutation in only one gene [31,39,42–47].

Many nevi, including common nevi, harbour the V600E mutation in the v-raf murine sarcoma viral oncogene homolog, B1 (*BRAF*) [48]. V600E was observed in 60% of all melanomas [49]. BRAF can regulate melanoma proliferation through MITF [50] that is a transcription factor and regulator of pigmentation.

Even though high melanoma risk alleles clearly exist in e.g. *CDKN2A*, it is speculated if the melanoma risk might be attributable to combinations of low to moderate risk alleles in e.g. *MC1R*, *TYR*, and *ASIP* [51].

In this study, we investigated the associations between SNPs in pigmentary genes and (1) quantitative skin colour measurements in 246 healthy, light skinned Danish individuals, (2) total nevi, (3) total atypical nevi count, and (4) the FAMMM syndrome in 116 Danish individuals at increased risk of developing malignant melanoma.

## Results

### Associations between quantitative skin colour and genetic markers

**Fig 1:**
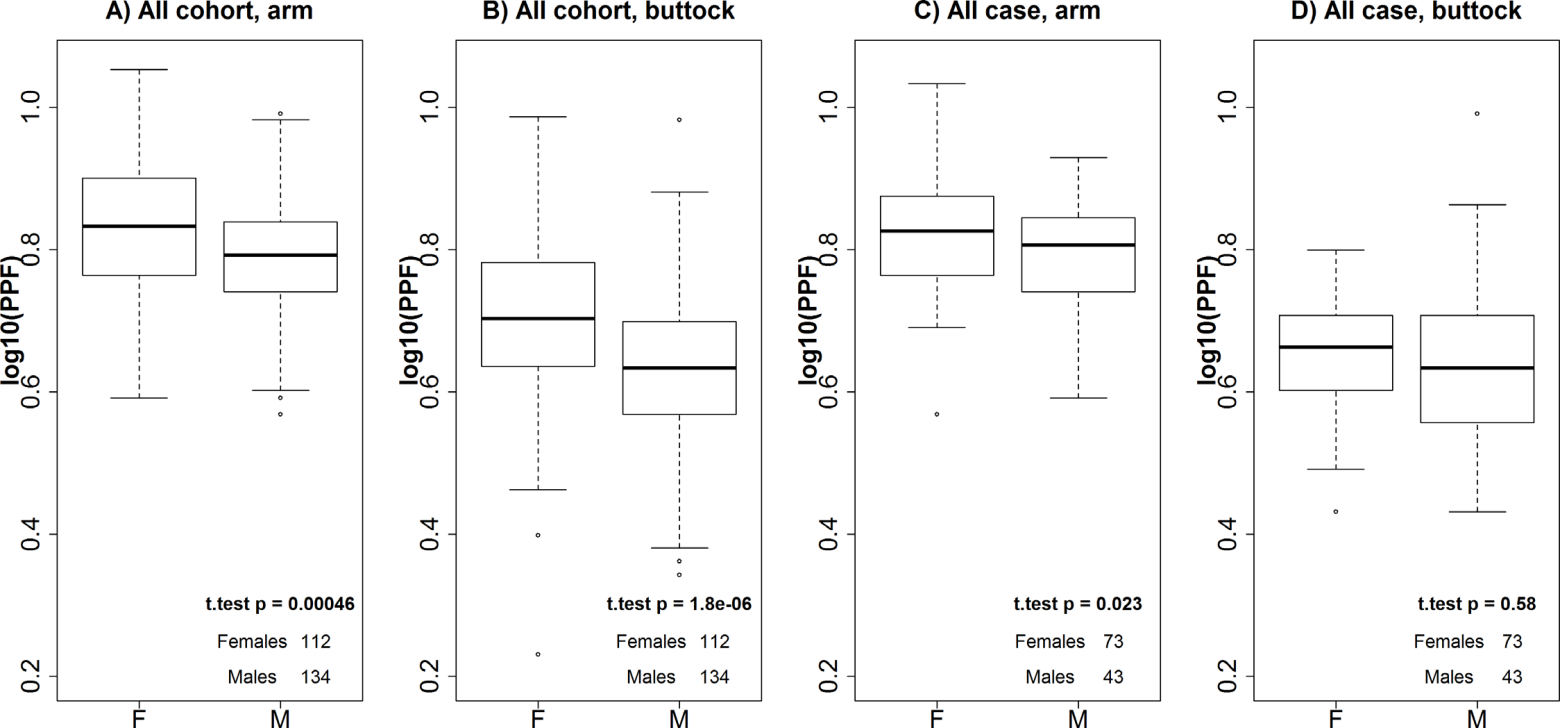
Comparison between skin color measurements in females and males. Arm (A) and buttock (B) measurements in females and males of the cohort. Arm (C) and buttock (D) measurements of female and male cases. P-values were calculated using Welch’s two sample t-test.

**Fig 2:**
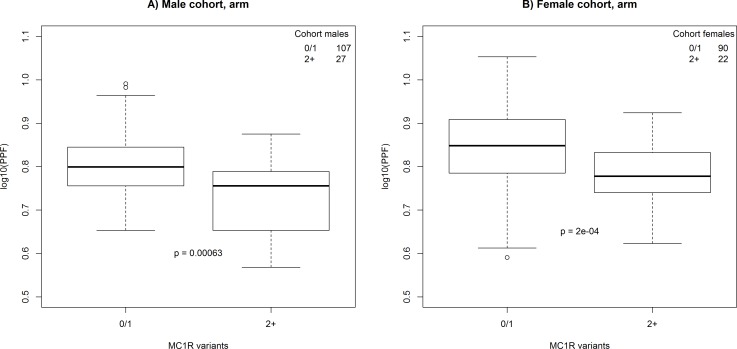
Comparison between skin colour measurements of the arm in male and female cohort individuals with ≤1 or ≥2 *MC1R* variants. P-values were calculated using Welch’s two sample t-test.

The Pearson correlation coefficient between logarithmic transformed arm and buttock PPF measurements was 0.51 (r^2^ = 0.26, data not shown). Buttock PPF measurements were statistically significantly lower than those of arm measurements for both genders in the cohort group (p<2.3x10^-16^). The skin was signifcantly darker in females than in males among the cohorts at both measurement sites (p<0.001, Figs 1A and 2B). Furthermore, female cases had statistically significantly higher arm PPF measurements than male cases (Fig 1C). However, we did not detect any statistically significant difference between the buttock PPF measurements of males and females in the case group (Fig 1D). Female cases had significantly lighter skin on the buttock than female cohorts (p = 0.0004). However, this tendency was not observed in males (p = 0.7), and there was no significant difference in the pigmentation levels on the arms between cases and cohorts in either sex (p = 0.8 and p = 0.9 in females and males, respectively).

### Influence of genetic variations, gender, age, and month of measurement on PPF

Individuals from the cohort group with two or more *MC1R* variants (missense, p.TYR152*, or p.Asn29Glnfs*14, S2 Table) had statistically significantly lower PPF measurements of the arm (Fig 2) than those having one or no *MC1R* variant. We did not observe this relationship for buttock measurements (data not shown). We performed multi linear regression model analysis of the cohorts (n = 246) with log10(PPF) as response and gender, age, month of measurement, 22 SNPs with sufficient observations, and *MC1R* variants as explanatory variables (Pane 1 S3 Table and Pane 2 S3 Table). For arm measurements, we only observed grouped *MC1R* variants to deviate statistically significantly (p<0.01). The *MC1R* association was not observed when investigating a similar multi linear regression model with buttock PPF as response. Among the tested variables, only gender was statistically significantly associated (p<0.01) with the buttock PPF.

### Associations with nevi and FAMMM

We evaluated the logistic regression model including age, gender, 22 SNPs, and *MC1R* for association with nevi count (Pane 3 S3 Table). A statistically significant association with total nevi count prior to correction for multiple testing was observed for the G and AG genotypes of rs6475555 on chromosome 9 (non-corrected p = 0.004 (corrected 0.18) and p = 0.005 (corrected 0.24), respectively). None of the parameters were statistically significantly associated with the total nevi count after correcting for multiple testing.

When evaluating a logistic regression model with atypical nevi count as response (Pane 4 S3 Table), we observed statistically significant association with age (p<0.05). Healthy cohorts had a mean age of 45.1, years and cases with atypical nevi had a mean age of 38.4 years, which may explain the statistically significant results of the model. The G and GA genotypes of rs1015362 in *ASIP* were statistically significantly associated with the atypical nevi count prior to correction for multiple testing (non-corrected p = 0.008 (0.36 corrected) and p = 0.003 (0.13 corrected), respectively).

When performing the logistic regression model including FAMMM (Pane 5 S3 Table), we observed a statistically significant association with age. This was most likely caused by the fact that patients with FAMMM had a mean age of 36.9 years, whereas the healthy cohorts had a mean age of 45.1 years. We did not observe any statistically significant association between the 22 SNPs or *MC1R* with the FAMMM syndrome, allthough that we prior to Bonferoni correction observed statistically significant associations with the vitamin D receptor (*VDR*) SNP rs1544410 G and the agouti signalling protein (*ASIP*) SNP rs1015362 G (non-corrected p = 0.007 (0.33 corrected) and p = 0.009 (0.43 corrected), respectively).

## Discussion

The measurements of pigmentation levels demonstrated that the buttock area was statistically significantly less pigmented than the upper-inner arm area. This implies that the buttock is a better site for measuring constitutive pigmentation levels than the upper-inner arm, and that the upper-inner arm measurements often reflect the tanning response of the individuals. We observed a statistically significant difference of the logarithmic transformed PPF values between males and females of the cohort group on both arm and buttock. This observation supports previous observations in eye colour studies indicating that there may exist a yet unknown biological, sex-determined mechanism controlling pigmentation levels [52,53]. On the other hand, the darker skin pigmentation levels observed in females could also be an environmental effect due to e.g. different sun or clothing habits (Fig 1A and 1B). The individuals in the case group are expected to avoid extensive UV-exposure. In this group, females and males did not have statistically significantly different PPF values of the buttock (Fig 1D), and the effect of gender on the arm pigmentation was less pronounced (Fig 1C). This seemed to indicate that the observed gender effect on pigmentation wass caused by an environmental effect.

When performing multi linear regression analysis among the cohorts, we observed that individuals with two or more *MC1R* variants had statistically significantly lower PPF values of the arm than those with none or one *MC1R* variants. *MC1R* variants were not statistically significantly associated with PPF measurements on the buttock. This shows, in concordance with established genetic theories concerning *MC1R* (reviewed in [54]), that *MC1R* variants are involved in human pigmentation. Since *MC1R* variants are not statistically significantly associated with pigmentation measurements of the buttock, we hypothesize that the effect of *MC1R* variants on arm pigmentation is primarily reflecting an inability to tan when subjected to UVR. Two of the genotypes of rs6475555 on chromosome 9 were, prior to correction for multiple testing, statistically significantly associated with the total nevi count. This SNP was previously found to be associated with the total nevus count in a study comprised of twins from the UK and twins of European ancestry in Australia [55]. Falchi and colleagues hypothesized that the gene methythoiadenosine phosphorylase (*MTAP*) or the adjacent *CDKN2A* gene are involved in the nevus formation [55]. A SNP in *MTAP* was reported to be associated with an increased number of nevi [33] indicating the importance of the *MTAP* region in nevus formation.

Two of the genotypes of *ASIP* rs1015362 were, prior to correction for multiple testing, found to be statistically significantly associated with the atypical nevi count. *ASIP* was described as being associated with skin colour and melanoma risk [55].

Neither the tested SNPs nor *MC1R* was statistically significantly associated with FAMMM after correction for multiple comparisons. This most likely reflects that FAMMM is caused by defects in non-pigmentary systems, e.g. the retinoblastoma or P53 pathways [39,40]. It is possible that the atypical nevi observed in these individuals are by-products of signalling through MITF rather than being caused by a specific function of the pigmentary system.

## Material and Methods

### Samples and DNA purification

Blood samples from 362 unrelated Danish individuals were collected. A sample of 246 healthy individuals (cohorts) was collected at the Section of Forensic Genetics, Department of Forensic Medicine, Faculty of Health and Medical Sciences, University of Copenhagen, and at the Blood Bank, Glostrup Hospital. Blood samples from 116 individuals (cases) were collected at the Department of Dermatology, Bispebjerg Hospital, Copenhagen, Denmark. The case group contained individuals attending regular inspection at the Department of Dermatology at Bispebjerg Hospital due to an increased risk of developing malignant melanoma. Not all case group individuals had a definite diagnosis. All individuals in the case group were evaluated by a trained dermatologist. Nevi count (> 50 or<50) and atypical nevi count (> 5 or < 5) were evaluated by the Dermatologist. Atypical nevi are large nevi (>5mm in diameter) with irregular borders and colour variations. Cases were heterogeneous and presented multiple indications; 71 cases had more than 5 atypical nevi and 59 cases had more than 50 nevi. Fifty cases were diagnosed with FAMMM using the following criteria; 1) 100 or more melanocytic nevi, 2) one or more melanocytic nevi greater than or equal to 8mm in its largest diameter, and 3) one or more clinically atypical melanocytic nevi (clinically defined entities, >5 mm in diameter, with irregular pigmentation and an irregular or diffuse edge), according to the description defined in [56].

The individuals in the cohort group were asked to fill in a questionnaire and answer if they had more than five large nevi (>5mm) on their skin. Individuals in the cohort group that reported more than five large nevi were excluded from the study of atypical nevi and FAMMM, as these individuals may represent undiagnosed case individuals. Individuals in the cohort group that reported less than five large nevi were classified as belonging to the non-FAMMM and the low nevi count category groups. This left 159 cohort individuals (87 provided no nevi information) and 116 case group individuals for analysis of nevi and FAMMM and a total of 246 cohort individuals and 116 cases for analysis of quantitative skin colour (Table 1).

**Table 1:** Descriptive statistics of the participants. F: females. M: males. Sd; Standard deviation.

Gender	Status	No of individuals	Age mean (years)	Age sd (years)	log(PPF),arm, mean	log(PPF), arm, sd	log(PPF), buttock, mean	log(PPF), buttock, sd
F	Cohorts	112	41	11.5	0.83	0.091	0.70	0.11
F	Case	73	38	8.4	0.82	0.081	0.65	0.08
M	Cohorts	134	43	11.1	0.79	0.084	0.63	0.12
M	Case	43	41	9.0	0.79	0.077	0.64	0.11

DNA was purified from 200μL whole blood using the DNA Blood Mini Kit (Qiagen) as recommended by the manufacturer. DNA was eluted in 50μL AE Buffer (Qiagen). Blood samples were collected between March 2010 and January 2012. Samples were grouped by the month of collection, regardless of year, in order to analyse the possible seasonal variation in quantitative skin reflectance measurements.

The study was approved by the Ethical Committee of the Capital region of Denmark, H-4-2009-125 and M-20090237. All participants provided written informed consent. All individuals included in the study reported Scandinavian parental origin. Individuals did not report regular use of tanning beds.

### Skin reflectance measurements

The quantitative skin reflectance measurements were performed using a UV-Optimize Scientific 555 (Chromo Light APS, Espergærde, Denmark). The instrument is a non-invasive spectrophotometer that calculates the reflectance of skin redness and pigmentation from the measured area in percentage [57]. The instrument was calibrated with a white standard (ISO 2469).

The reflectance of a wavelength at 555nm measures the haemoglobin content and is scaled as 0%-100% skin redness. Null percent skin redness corresponds to skin without blood, and 100 percent corresponds to highly vascular skin lesions. The reflectance of 660 nm indicates the melanin content termed skin pigmentation when the skin redness is eliminated. A null percentage skin pigmentation measurement corresponds to the reflection of no pigmentation (pale), and a 100 percentage skin pigmentation measurement corresponds to the reflection of black skin [57]. The pigment protection factor (PPF) is a value for the protection against UVR provided by skin pigmentation and the top layer of epidermis (stratum corneum). The PPF can be used in parallel with the Fitzpatrick skin types [58]. Measurements were performed on the upper, inner arm and on the buttock in triplicates for each participant. The medians of each PPF triplicates were used for statistical analyses. All measured skin areas were free from nevi, freckles, tattoos, and hair.

### SNP typing

All 362 samples were typed for 32 SNPs, which were chosen based on their associations to various skin malignancies and skin colours (S1 Table), using the iPLEX^®^ Gold kit (Sequenom) (S1 Table). The PCR contained 2μL DNA, 0.5μL 10x Buffer, 0.8μL 25mM MgCl_2_, 0.1μL 25mM dNTP mix, 1.3μL 0.5μM primer mix (DNA Technology), 0.2μL 5U/μl HotStarTaq, and 1.1μL H_2_O. The PCR was performed in a GeneAmp^®^ PCR system 9700 thermal cycler (Applied Biosystems) with the following conditions: denaturation at 94°C for 2 min followed by 45 cycles of 94°C for 20 s, 62°C for 30 s, 72°C for 1 min, followed by 72°C for 3 min. The PCR products were treated with Shrimp Alkaline Phosphatase (SAP) (Sequenom) in a GeneAmp^®^ PCR system 9700 thermal cycler at 37°C for 40 min and 85°C for 5 min. The SBE reaction contained 8μl SAP treated PCR products and 2μl iPLEX^®^ mix (Sequenom). The iPLEX^®^ mix contained 0.2μL 10x iPLEX^®^ buffer, 0.2μL iPLEX^®^-Termination mix, 0.94μL primer mix (DNA Technology), 0.04μL iPLEX^®^ enzyme, and 0.62μL H_2_O.

The SBE reaction was performed in a GeneAmp^®^ PCR system 9700 thermal cycler with the following conditions: denaturation at 94°C for 30 s followed by 40 cycles of 94°C for 5 s, 52°C for 5 s and 80°C 5 s, 52°C for 5 s and 80°C for 5 s, 52°C for 5 s and 80°C for 5 s, 52°C for 5 s and 80°C for 5 s, 52°C for 5 s, 80°C 5 s, and 72°C for 3 min.

A total of 40μL of molecular grade water and ion exchange resin (Sequenom) was added to each sample. Samples were rotated for approximately 4 h and kept in the refrigerator for up to 4 days before spotting. The samples were spotted in duplicates using the RS1000 Nanospotter (Sequenom) and visualized on the MassARRAY^®^ analyzer 4 system (Sequenom) using the autorun settings.

Samples were analysed with Typer Analyzer 4 (Sequenom) and were autoclustered using a signal to noise ratio of 7. Clusterplots were visually inspected, and outliers were further investigated.

All samples were run in duplicates. Genotypes were compared between spots and duplicate typings as described in [53] and implemented with the statistic software R (R core team, version 2.11.0, URL http://www.R-project.org). The SNPs rs26722, rs28777, rs1426654, rs1800407, rs7495174, rs8059973, rs12203592, rs12913032, rs16891982 and rs36118030 all had minor allele frequencies (MAF)<0.01 and ≤4 observations, and were removed from the analysis.

### Sequencing of *MC1R*

The *MC1R* gene of all 362 individuals was sequenced. A region encompassing the *MC1R* exon and the promoter region was amplified in a single PCR amplicon of 1,963bp. Prior to library preparation, we employed a multiplexing approach using endonuclease digestion of the PCR product as previously described [59]. Libraries were prepared for sequencing with the TruSeq® DNA Sample Preparation kit (Illumina) and paired-end sequenced (2x250 cycles) on the Illumina Miseq platform using the Illumina MiSeq Reagent Nano kit v2 (500 cycles).

Illumina adaptors were trimmed using Flexbar [60]. The FASTQ files were aligned to the *MC1R* reference sequence assembly Feb.2009 GRCh37/hg19 (UCSC Genome Browser) with the Burrows-Wheeler Aligner (BWA) MEM algorithm [61] to generate BAM files. Variant calling was carried out using GATK ver. 2.6.5 [62]. Variants were accepted if they had a minimum coverage of 50. Variants, where two different alleles were observed, were accepted as heterozygote variant calls if the frequency of the minor variant allele was >0.15. Variants were analysed using Alamut Batch (Interactive Biosoftware, France). *MC1R* variants were grouped according to potential significance. All missense, non-sense, and frameshift mutations (S2 Table) were assigned to the *MC1R* variant group and analysed together (Table 2). Due to a low number of participants and a number of unclassified variants detected, the R/r system of MC1R mutation classification [63] was disregarded.

**Table 2:** Number of *MC1R* variants.

Group/*MC1R* variants	No of individuals	0	1	2	3
**Cohort**	246	89	108	49	0
**Cases**	116	36	65	13	2

### Statistical analyses

**Fig 3:**
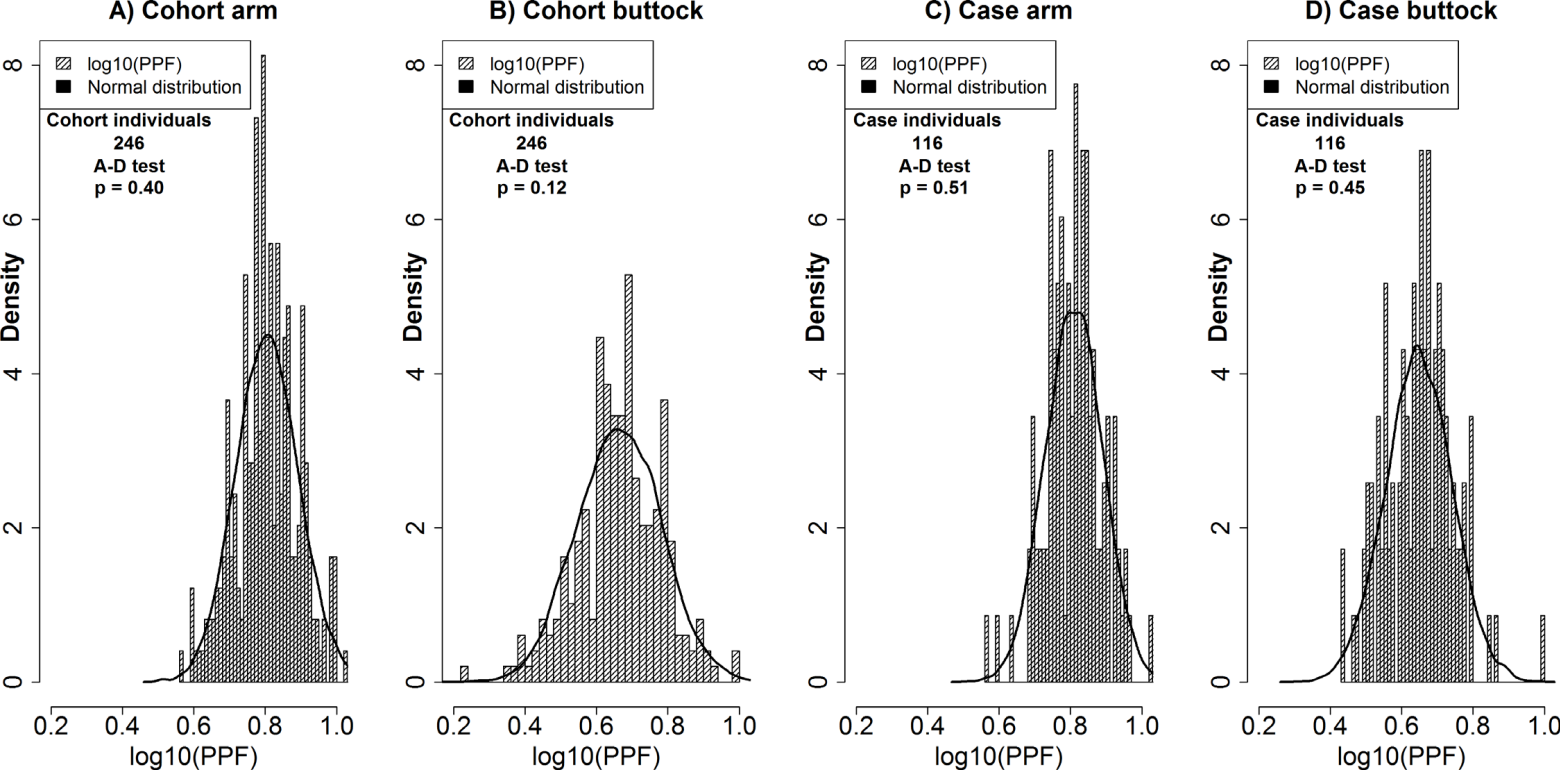
Distributions of arm (A) and buttock (B) measurements in the cohort. Distribution of arm (C) and buttock (D) measurements in the cases. The black line represents a simulated normal distribution with 10,000 observations with the same mean and standard deviation as that of the underlying data.

All statistic calculations were performed using R (R core team, version 3.1.1, URL http://www.R-project.org). The PPF data was log transformed as this gave the best approximation to the normal distribution. All PPF data groups passed the Anderson-Darling test for normality (Fig 3). Correlations between logaritmically transformed PPF measurements of arm and buttock areas were investigated using the Pearson correlation coefficient. Multi linear models were performed using the *lm* command of R. Models were performed with log10(PPF) as response and gender, age, month of measurement, 22 SNPs with sufficient observations (MAF>0.01), and grouped *MC1R* variants as explanatory variables (25 variables, 53 comparisons). Logistic regression models were performed using the *glm* command of R. Models were constructed for age, gender, 22 SNPs and as *MC1R* to test for association with grouped nevi count or FAMMM (24 variables, 48 comparisons). Welch’s two sample t-test was used due to differences in sample sizes. P-values were corrected for multiple comparisons using Bonferroni correction.

## Supporting Information

S1 Table SNPs, PCR and SBE primers for the iPLEX Gold reactions. (DOCX)

S2 Table Overview of coding MC1R variants. (DOCX)

S3 Table Linear and Generalized linear models. (XLSX)
