# Supplementary material for: Pigmentary Markers in Danes – Associations with Quantitative Skin Colour, Nevi Count, Familial Atypical Multiple-Mole, and Melanoma Syndrome
Source: PLoS One. 2016 Mar 3;11(3):e0150381. doi: 10.1371/journal.pone.0150381 (PMC4777533; doi:10.1371/journal.pone.0150381)
Supplement: S1 Table — (DOCX) [file pone.0150381.s001.docx]

Table S1. SNPs, PCR and SBE primers for the iPLEX Gold reactions.

| SNP_ID | Concentration PCR primers | 2^nd^ PCRP | 1^st^ PCRP | Concentration SBE µM | SBE primer | Gene/locus | References |
| --- | --- | --- | --- | --- | --- | --- | --- |
| rs26722 | 500nm each | ACGTTGGATGTTGCCAGCTCTGGATTTACG | ACGTTGGATGGTCATCAGATGGAATGTACG | 11.6 | CCGTAACCATTTTTAACTTTCT | *SLC45A2* | [17] |
| rs28777 | 500nm each | ACGTTGGATGCAAGAGTCGCATAGGACAGG | ACGTTGGATGGCTTCCACTCAGTTGATTTC | 7 | CCCATCCACTCAGAG | *SLC45A2* | [10] |
| rs199355 | 500nm each | ACGTTGGATGCTGACCATGCTGTCCAGTTG | ACGTTGGATGACTCATGGCTGGTTTCTATC | 9.4 | TGTCCAGTTGTAAACATA | *GPNMB* | [60,61] |
| rs258322 | 500nm each | ACGTTGGATGGAAACCTGTTCAGGAACTTC | ACGTTGGATGACCGGATGCTTACGTTTACC | 14 | TTCATGATGTAGTTATCACAAAC | *MC1R/CDK10* | [8] |
| rs401681 | 500nm each | ACGTTGGATGGCCAGAAAGCTGCTTCACAC | ACGTTGGATGATGCATAGTGGGCAGAAAAC | 11.6 | TATCGGCTTCACACCATGAT | *CLPTM1L* | [62,63] |
| rs1015362 | 500nm each | ACGTTGGATGTTGCTGAACAAATAGTCCCG | ACGTTGGATGAAGTGTGTACTGTGTGTCTG | 9.4 | AGGAGATGAAAACATCTCA | *ASIP* | [12,64] |
| rs1042522 | 500nm each | ACGTTGGATGAGACCCAGGTCCAGATGAAG | ACGTTGGATGGGTGTAGGAGCTGCTGGTG | 9.4 | CGCCAGAGGCTGCTCCCC | *TP53* | [65] |
| rs1042602 | 500nm each | ACGTTGGATGGGTGCTTCATGGGCAAAATC | ACGTTGGATGGTGTCAATGGATGCACTGCT | 11.6 | ACCAATGTCTCTCCAGATTTCA | *TYR* | [26] |
| rs1393350 | 500nm each | ACGTTGGATGATGCGTGCATATCCACCAAC | ACGTTGGATGGGAAGGTGAATGATAACACG | 7 | TCCCTTCTCTGCAAC | *TYR* | [8,66] |
| rs1426654 | 500nm each | ACGTTGGATGAATTCAGGAGCTGAACTGCC | ACGTTGGATGTTCAGCCCTTGGATTGTCTC | 11.6 | CCCGCTGCCATGAAAGTTG | *SLC24A5* | [67–69] |
| rs1544410 | 500nm each | ACGTTGGATGTAGATAAGCAGGGTTCCTGG | ACGTTGGATGAGCCCAGTTCACGCAAGAG | 7 | CCACAGACAGGCCTGC | *VDR* | [33,70] |
| rs1800407 | 500nm each | ACGTTGGATGTGATGATCATGGCCCACACC | ACGTTGGATGACTCTGGCTTGTACTCTCTC | 7 | CGGCCCACACCCGTCCC | *OCA2* | [11] |
| rs2031526 | 500nm each | ACGTTGGATGGGGTCAAATGTCATTTGAGG | ACGTTGGATGTGCCCAGCCCAATGATACAC | 9.4 | ATCATTTGAGGGTAGGAA | *DCT* | [71] |
| rs2228570 | 500nm each | ACGTTGGATGTGGCCTGCTTGCTGTTCTTA | ACGTTGGATGAAGTCTCCAGGGTCAGGCA | 11.6 | GCTGGCCGCCATTGCCTCC | *VDR* | [33,70] |
| rs2276288 | 500nm each | ACGTTGGATGATGGTGACAGTGGGCATGAC | ACGTTGGATGGCCAACGGCATCAATGAGAG | 14 | TAAAGTGGGCATGACGTACACAC | *MYO7A* | [11] |
| rs2284063 | 500nm each | ACGTTGGATGTGCCTGATTACACTTTCCCC | ACGTTGGATGAGGAGTTTTGCTGTGGTGAG | 14 | CATTAAACTTTCCCCATTCGCTA | *PLA2G6* | [59] |
| rs2762464 | 500nm each | ACGTTGGATGCTGTTGACAAATATCCATGA | ACGTTGGATGCTTAAAATACATGAATGGGC | 11.6 | AAATATCCATGAATGGAAATG | *TYRP1* | [72] |
| rs3733542 | 500nm each | ACGTTGGATGTGAAGTTGTCTTTGGCAAGG | ACGTTGGATGAAGTGACGTCTGGTCCTATG | 14 | AGGATCATTTTACCTAAAGAGAA | *KIT* | [11] |
| rs4778241 | 500nm each | ACGTTGGATGTGCAATTGTTGGCTGGTAGT | ACGTTGGATGAAATTGTACAGCCACTCTGG | 7 | TGCTGGTAGTTGCAATT | *OCA2* | [11] |
| rs4821767 | 500nm each | ACGTTGGATGTTCTACAGCCAAGTGGAAAC | ACGTTGGATGTGGGAAAGCCACTCTCCCT | 14 | CTCCATCCAAGTGGAAACTATCTT | *Chr 22* | [59] |
| rs4911414 | 500nm each | ACGTTGGATGGGCCCCCAGTCTCTTTTTG | ACGTTGGATGGGCAACTAGAGAAAAGCATC | 14 | CTTATTTGCTGAGAAATTCATT | *ASIP* | [12] |
| rs4911442 | 500nm each | ACGTTGGATGCTTGAGGTAACCTGTAAATGG | ACGTTGGATGCTGAACTGTAGCTAATGAAC | 14 | ATGGAAATGGTAGTACCAGAAT | *ASIP* | [10] |
| rs6475555 | 500nm each | ACGTTGGATGGAAAGAACTGACACCAGTCC | ACGTTGGATGGAATGAGTTAGGGAGGATGC | 14 | CATCCTACTGAAACTATTCC | *Chr 9* | [59] |
| rs7495174 | 500nm each | ACGTTGGATGTTAGGAAGCAAGGCAAGTTC | ACGTTGGATGTAGGTCGGCTCCGTCGCAC | 11.6 | CCTTAAGTTCCCCTAAAGGT | *OCA2* | [15,26] |
| rs8059973 | 500nm each | ACGTTGGATGGGCAGGTGGTTCTGTGTTAA | ACGTTGGATGTTCCCGAGTAGCTGCCACAC | 14 | GGGTCTGTGTTAATAATGACAGCT | *MC1R/DBNDD1* | [8] |
| rs11614913 | 500nm each | ACGTTGGATGGGTAGTTTCATGTTGTTGGG | ACGTTGGATGTCGACGAAAACCGACTGATG | 7 | TCGGCAACAAGAAACTG | *MIR196A2* | [73] |
| rs12203592 | 500nm each | ACGTTGGATGGTTTCATCCACTTTGGTGGG | ACGTTGGATGGTCATATGGCTAAACCTGGC | 9.4 | GGTGGGTAAAAGAAGG | *IRF4* | [66,74] |
| rs12623857 | 500nm each | ACGTTGGATGCTTCCCACAGTGTCCACTC | ACGTTGGATGGCAGCGGCCTGCCGTTGAT | 9.4 | CCTCAACCAGCTCGGCGG | *PAX3* | [11] |
| rs12896399 | 500nm each | ACGTTGGATGTCTGGCGATCCAATTCTTTG | ACGTTGGATGAGGAAGGTTAATCTGCTGTG | 14 | GTAGGTCAGTATATTTTGGG | *SLC24A4* | [26] |
| rs12913832 | 500nm each | ACGTTGGATGCCCCTGATGATGATAGCGTG | ACGTTGGATGTCTGTGTCTGATCCAAGAGG | 11.6 | AGAGAGTGCAGAACTTGACA | *HERC2* | [75] |
| rs16891982 | 500nm each | ACGTTGGATGCACAGAGTTTCTCATCTACG | ACGTTGGATGAAAGTGAGGAAAACACGGAG | 7 | TGGATGTTGGGGCTT | *SLC45A2* | [10,13,17,63,66,76] |
| rs36118030 | 500nm each | ACGTTGGATGAGGGAGGATTCGCTAACAAG | ACGTTGGATGAGCTTCCAAAACAAGCAGCC | 9.4 | CAGCTAACAAGTGTGCTC | *MITF* | [11,77–80] |
