# Supplementary material for: Pigmentary Markers in Danes – Associations with Quantitative Skin Colour, Nevi Count, Familial Atypical Multiple-Mole, and Melanoma Syndrome
Source: PLoS One. 2016 Mar 3;11(3):e0150381. doi: 10.1371/journal.pone.0150381 (PMC4777533; doi:10.1371/journal.pone.0150381)
Supplement: S2 Table — (DOCX) [file pone.0150381.s002.docx]

Table S2 Overview of coding MC1R variants.

| Chr. | Position | dbSNP | Coding effect | Amino acid change | RHC* | MAF |
| --- | --- | --- | --- | --- | --- | --- |
| 16 | 89986608 | rs2228478 | synonymous | Thr314Thr |  | 0.09945 |
| 16 | 89986546 | rs1805009 | missense | Asp294His | R | 0.01519 |
| 16 | 89986503 | rs202197434 | missense | Asn279Lys |  | 0.00414 |
| 16 | 89986458 | rs181269865 | synonymous | Ile264Ile |  | 0.00138 |
| 16 | 89986421 |  | missense | Leu252His |  | 0.00138 |
| 16 | 89986365 | rs146544450 | synonymous | Gln233Gln |  | 0.00276 |
| 16 | 89986268 |  | missense | Val201Gly |  | 0.00829 |
| 16 | 89986241 |  | missense | Leu192Arg |  | 0.00138 |
| 16 | 89986154 | rs885479 | missense | Arg163Gln | r | 0.03039 |
| 16 | 89986144 | rs1805008 | missense | Arg160Trp | R | 0.06492 |
| 16 | 89986130 | rs1110400 | missense | Ile155Thr | R | 0.05525 |
| 16 | 89986122 | rs201326893 | nonsense | Tyr152* |  | 0.00138 |
| 16 | 89986117 | rs1805007 | missense | Arg151Cys | R | 0.05525 |
| 16 | 89986091 | rs11547464 | missense | Arg142His | R | 0.00414 |
| 16 | 89985940 | rs2228479 | missense | Val92Met | r | 0.06354 |
| 16 | 89985918 | rs1805006 | missense | Asp84Glu | R | 0.01243 |
| 16 | 89985844 | rs1805005 | missense | Val60Leu | r | 0.13398 |
| 16 | 89985778 | rs200050206 | missense | Val38Met |  | 0.00276 |
| 16 | 89985750 |  | Frameshift | Asn29Glnfs*14 |  | 0.01243 |
|  |  |  | Any missense, nonsense or frameshift variant |  |  | 0.24033 |
|  |  |  | Any two missense, nonsense or frameshift variant |  |  | 0.08425 |
|  |  |  | Any three missense, nonsense or frameshift variant |  |  | 0.00276 |
|  |  |  | Any R variant |  |  | 0.12983 |
|  |  |  | Any two R variant |  |  | 0.01381 |

*Classified according to [25]
